# The effect of lead on the process of spermatogenesis in sex glands of male albino rats

**DOI:** 10.14202/vetworld.2016.1129-1134

**Published:** 2016-10-23

**Authors:** Olga Sergeevna Shubina, Natalia Anatolievna Dudenkova

**Affiliations:** Federal State Budgetary Educational Institution of Higher Education, M. E. Evseviev Mordovian State Pedagogical Institute, Saransk, Russia

**Keywords:** epididymis (appendage testis), lead acetate, seminal glands (testis), Sertoli cells, spermatogenesis, spermatogenic cells

## Abstract

**Aim:**

The aim of this study was to investigate the morphological and functional features of the process of spermatogenesis in the testes of male albino rats when exposed to lead acetate.

**Materials and Methods:**

Using histological, morphometric and statistical methods explored the effects of lead acetate on the process of spermatogenesis in the testes of male albino rats. Investigations were carried out using a digital microscope Axio Imager. M2 and automatic cell counter Countess™.

**Results:**

It is shown that when exposed to lead acetate a decrease in the production of all populations of spermatogenic cells, decreased spermatogenesis index and an index of relaxation (tension spermatogenesis), the increase in the index of ripening, index meiotic activity and germinative index, which indicates a decrease in the functional activity of the testes. On preparations, it is possible to see that after the influence of acetate of lead the head of spermatozoa becomes more roundish, breaks of tails observed.

**Conclusion:**

Lead acetate reduces the productivity of the seminal glands, which leads to the decrease of the concentration of spermatozoa, and their viability. The results of the studies suggest a negative impact of lead acetate in the course of the process of spermatogenesis in the testes of male white rats.

## Introduction

With the onset of puberty in male sex glands (testes) begins the process of maturation of male germ cells - spermatogenesis, which is extremely sensitive to the damaging effects, including the effects of heavy metals, which is lead [1-5].

However, experimental data on the influence of heavy metals on the testes very little, and the available data are rather contradictory. There is little empirical data on the impact of lead on the course of the process of spermatogenesis, its functional changes, and also not clear what level of gametogenesis in quantitative terms suffers more [6-8].

The aim of this study was to investigate the morphological and functional features of the process of spermatogenesis in the testes of male albino rats when exposed to lead acetate.

## Materials and Methods

### Ethical approval

The animals were killed by decapitation under ether anesthesia with chloroform (1:1) in compliance with the principles of humanity as set out in the directives of the European Community (86/609/EES) and the Declaration of Helsinki and in accordance with the rules of carrying out the works using experimental animals.

### Animals

The pubescent outbred albino male rats weighing 200-250 g were used as a biological test object.

### Experimental design

Seminal glands were used as a trial material for study. The experiment was conducted during the year in the premises with air temperature 22-25°C and a relative humidity 67-70%. In line with the research objectives, the animals were divided into two groups. The control group of animals was rats contained on the common regime of the vivarium. Experimental group included animals that received within 7 days of oral acetate lead Pb(CH_3_COO)_2_×3H_2_O in intermediate toxicity dose of 45 mg/kg/day (in terms of lead). For histological study tissue samples, seminal glands were fixed in 10% solution of the neutral formalin. Preserved samples after rinsing in running water were dehydrated by placing in alcohols of increasing concentration and embedded into paraffin according to the conventional methodology. Histological cross sections of seminal glands were prepared 10-15 µ thick, stained with hematoxylin-eosin and examined by a digital microscope Axio Imager.M2 with the image analysis software AxioVision SE64 Rel. 4.8.3 and ZEN 2011.

Morphometric measurements were performed with a zooming of 40×10. The preparations were photographed with a digital camera AxioCam MRc5 (ZEISS, Japan), and then the images were processed in the Adobe Photoshop Elements 11. Resolution of the resulting images was 1300×1030 pixels.

Using histological research methods and morphometric analysis studied the structural and quantitative changes of various kinds of the spermatogenic cells in normal conditions and after 7 days of exposure of lead acetate Pb(CH_3_COO)_2_×3H_2_O.

### Tests, procedures, etc.

On the basis of morphometric data of the testes were counting the number of informative parameters, characterizing the state of spermatogenesis:

1. Spermiogramma – Percentage distribution of spermatogenic epithelium cells [9].
2. Index of spermatogenesis – Ratio of the sum of all the layers of cells counted in one tubule to the number of counted tubules.
  Spermatogenesis index was calculated by the formula: Is=∑a/N, Where a – is the number of layers selected in each tubule (the first layer is spermatogonia, the second layer is spermatocytes, the third layer is spermatids, and the fourth layer is spermatozoons); N – is the number of counted tubules [10].
3. Index of relaxation (tension of spermatogenesis) – The ratio of the sum of all the spermatogenic cells to the amount of Sertoli cells [9].
4. Index of ripening – The ratio of young (spermatogonia and spermatocyte) and mature forms of spermatogenic epithelium (spermatids and spermatozoa).
5. Index meiotic activity – Ratio of meiotic cells (spermatocytes) to a sum the remaining germ cells.
6. Germinative index – The ratio of spermatogonia to a sum the Sertoli cells [11].

To determine the index of relaxation and germinative index counted the number of Sertoli cells in the spermatogenic epithelium convoluted seminiferous tubule testes using a digital microscope Axio Imager.M2 (ZEISS, Japan) with software for image analysis AxioVision SE64 Rel. 4.8.3 and ZEN 2011 with an increase of 40×10 [12].

It is known that a suspension of spermatozoa is highly sensitive to toxic substances [13-15], so one of the objectives of our study was to investigate the morphological and functional changes in spermatozoa of male albino rats when exposed to lead acetate.

Effects of lead acetate on the suspension of spermatozoa of male albino rats were evaluated on the following parameters:

1. The total concentration of spermatozoa;
2. The concentration of live spermatozoa;
3. Concentration of dead spermatozoa;
4. Spermatozoa viability (% living cells of their total number).

To determine the above-mentioned indicators of the tail of the longitudinally dissected and freed from fat appendage testis (epididymis) received 1 ml of spermatozoa suspension was diluted with saline (1:4), picked up 1 ml of a mixture of which will make a smear on a glass slide, stained with trypan blue and examined using an automatic cell counter Countess™ (Invitrogen, USA) with an increase of 100×2.3.

Living cells trypan blue stains on the edges of the dead - uniformly throughout the cell [16,17].

To analyze the quality spermatozoa smear spermatozoa suspension was examined using a digital microscope Axio Imager.M2 (ZEISS, Japan) with an increase of 40×10.

### Statistical analysis

Statistical processing of digital data was performed using the FStat and Excel program codes. Testing of statistical hypothesis was carried out by Student’s t-test. When testing statistical hypotheses, the accepted significance points were p≤0.05.

## Results

Histological examination of the testes white rats showed that in the first outer layer spermatogenic epithelium in the tortuous seminiferous tubules are lying on the basal membrane of spermatogonia with dark optically dense core and narrow bezel cytoplasm.

Closer to the center of the tubule located spermatocytes. These large cells with a large nucleus and cytoplasm of a wide rim having a rounded shape.

The innermost layer of convoluted tubules is spermicide, small with a light nucleus of the cell, lying in rows. Early spermatids rounded shape with a spherical nucleus is in the middle layers of spermatogenic epithelium. Late spermatids are in the layer adjacent to the lumen of the tubule, have an elongated shape. Some late spermatids detected flagellum.

**Figure-1:**
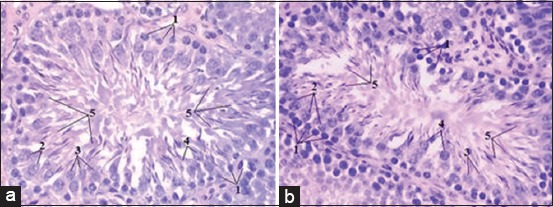
Convoluted seminiferous tubule. Stained with hematoxylin and eosin (40×10): (a) Control, (b) experiment, 1 – spermatogonia, 2 – spermatocytes, 3 – early spermatids, 4 – late spermatids, and 5 – spermatozoa.

In some tubules are seen formed spermatozoa. Their dark elongated head focused on the periphery of the tubule and tails hanging in the lumen of the tubule. Spermatozoa in the lumen of convoluted tubules groups are located in the amount of 6-8 around the contour of the lumen (Figure-1a).

**Figure-2:**
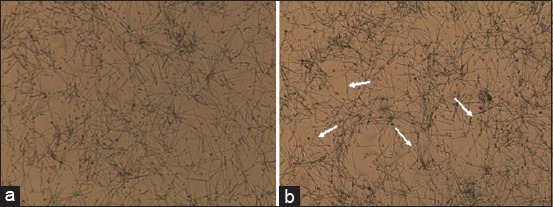
Spermatozoa of male albino rats when viewed under an automatic cell counter Countess™ (Invitrogen, USA). Dye trypan blue (100×2.3): (a) Control, (b) experiment (arrows indicate breaks tails and spermatozoa agglutination).

However, a closer examination of spermatozoa using a digital microscope Axio Imager.M2 at increase of 40×10 established that the head has the shape of a hook (Figure-2a).

**Figure-3:**
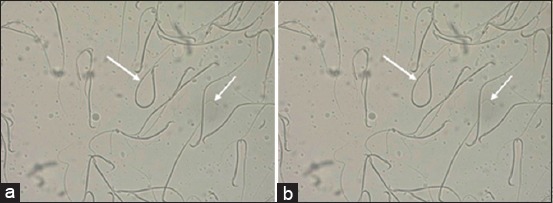
Spermatozoa of male albino rats (40×10): (a) Control, (b) experiment (arrows indicate breaks tails and spermatozoa agglutination).

After research on the impact of lead acetate in the spermatozoa suspension revealed that in the control group of animals is cloudy or milky white, has a thick consistency. The observed high concentration of spermatozoa in the mix (Figure-3a).

**Figure-4:**
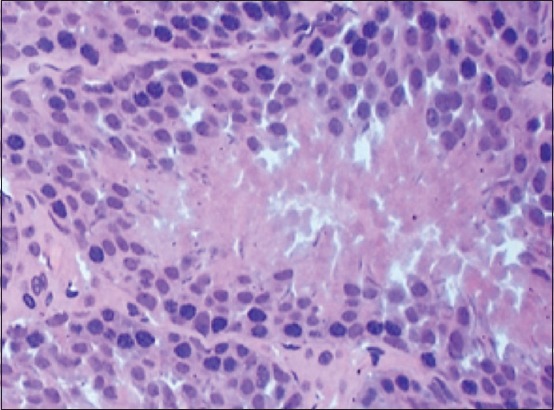
Convoluted seminiferous tubule (experiment). It is clear absence of spermatozoa in the lumen of the tubule. Stained with hematoxylin-eosin (40×10).

Histological research of drugs testes white rats after 7 days of exposure to lead acetate showed that spermatogonia, compared to control, are smaller. Spermatocytes become oval, rarely spherical. Early and late spermatid practically does not differ. They mainly oval, their nuclei are displaced in the center of the cell (Figure-1b). Noted a single location of spermatozoa in the lumen of the tubule. Found convoluted tubules, in the lumen of which were absent spermatozoa (Figure-4).

Observed chaotic arrangement of spermatozoa in the lumen of the tubule. Change in the shape of the spermatozoa head. She has a more rounded form. On history path observed on cliffs tails and of spermatozoa agglutination (Figure-2b).

After 7 days of exposure to lead acetate noted that the pick of spermatozoa suspension becomes more transparent color and less viscous (Figure-3b).

Morphometric studies have shown that in the experimental group of animals compared to the control, there is a decrease in the number of spermatogonia, spermatocyte, spermatids and spermatozoa, respectively, on 6.31% (p≤0.05), 8.43% (p≤0.05), 17.36% (p≤0.05) and 26.70% (p≤0.05) (Table-1).

**Table-1:** Quantitative and percentage change of the different types of spermatogenic cells in the tortuous seed tubules of the testes of male white rats under the influence of lead acetate.

Indicators	Control		Experiment	

	The number of cells in the tortuous seminiferous tubule	Percentage of total number of spermatogenic cells	The number of cells in the tortuous seminiferous tubule	Percentage of total number of spermatogenic cells
Spermatogonia	52.44±1.46	12.12±2.71	49.44±1.30*	16.59±2.56*
Spermatocytes	40.80±1.97	9.43±1.61	37.36±1.71*	10.03±2.37*
Spermatid	34.80±1.52	8.04±1.20	28.76±1.31*	7.49±1.35*
Spermatozoa	304.52±13.14	70.41±4.14	223.20±31.02*	65.89±5.20*

* p≤0.05 versus control animals

**Figure-5:**
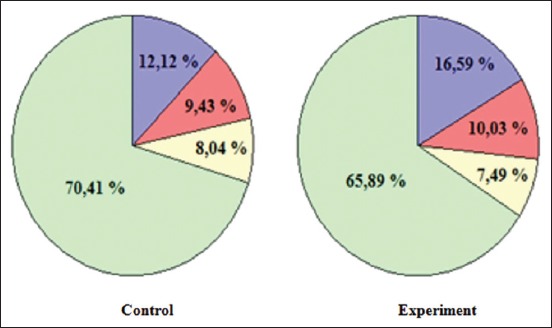
Spermiogramma male white rats: 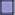 – Spermatogonia, 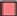 – Spermatocytes, 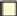 – Spermatid, 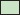 – Spermatozoa.

In the study spermiogram, male albino rats found that when exposed to lead acetate reduced the percentage of more mature forms of the spermatogenic cells - spermatids and spermatozoa and increases the percentage of spermatogonia and spermatocyte (Figure-5).

To determine the index of relaxation and its germinative index calculated the number of Sertoli cells in the spermatogenic epithelium of convoluted tubules seminal glands. Morphometric studies have shown that in the experimental group of animals compared to the control, there was a significant decrease in the number of Sertoli cells with at 23.84±3.16 to 18.48±2.52, i.e., to 22.48% (p≤0.05).

After investigating, the marked decrease of the index of spermatogenesis and index relaxation (tension of spermatogenesis), compared with the control, respectively, 10.24% (p≤0.05), 4.46% (p≤0.05), indicating a decrease of functional activity of the seminal glands.

Simultaneously, the index is increased maturation index meiotic activity and germinative index, compared with the control, respectively, at 20.00% (p≤0.05), 23.08% (p≤0.05) and 31.79% (p≤0.05), suggesting the predominance young cells of more mature, and delay maturation of male germ cells (Table-2).

**Table-2:** The change of the functional activity of the testes of male white rats under the influence of lead acetate.

Indicators	Control	Experiment
Index of spermatogenesis	3.32±0.15	2.98±0.12*
Index of relaxation (tension of spermatogenesis)	18.14±1.72	17.33±1.02*
Index of ripening	0.28±0.01	0.35±0.04*
Index meiotic activity	0.10±0.01	0.13±0.01*
Germinative index	2.21±0.17	3.24±0.36*

* p≤0.05 versus control animals

**Figure-6:**
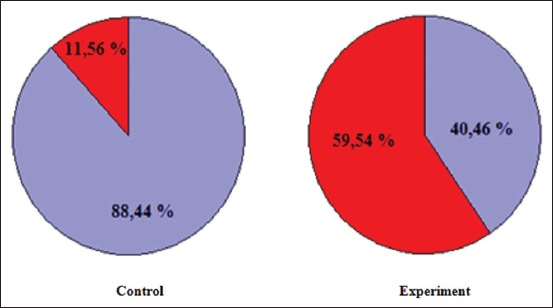
Suspension of spermatozoa of male albino rats: 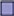 – Live spermatozoa, 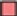 – Dead spermatozoa.

Conducted studies on the viability of spermatozoa showed that in the experimental group of animals compared to the control, there is a reduction of the total concentration of spermatozoa in 1 ml of suspension, the concentration of live spermatozoa and their viability respectively on 50.63% (p≤0.05), 77.41% (p≤0.05), and 53.05% (p≤0.05). Simultaneously, there is an increase in the concentration of dead spermatozoa on 60.68% (p≤0.05) (Table-3 and Figure-6).

**Table-3:** Quantitative and qualitative indicators of productivity of the testes of male white rats.

Indicators	Control	Experiment
The total concentration of spermatozoa, ×10^7^/ml	7.96±0.45	3.93±0.11*
The concentration of live spermatozoa, ×10^7^/ml	7.04±0.12	1.59±0.09*
Concentration of dead spermatozoa, ×10^7^/ml	0.92±0.07	2.34±0.14*
Spermatozoa viability, %	88.62±3.48	35.57±2.75*

* p≤0.05 versus control animals

## Discussion

For analysis of cytological profile of spermatogenesis, we were counting the total number of spermatogenic cells in Priest River sections of convoluted seminiferous tubules, as well as the counting of certain types of spermatogenic cells: Spermatogonia of various degrees of maturity, and their total number of primary and secondary spermatozoa early and late spermatids spermatozoa.

The study of the content of spermatogenic cells in seminiferous convoluted tubule has allowed to establish that in experimental animals in the convoluted seminiferous tubules of the observed increase in the percentage of spermatogonial cells in the seminiferous epithelium of the general number of germ cells at 4.45% compared with controls, suggesting enhanced proliferation of gonocytes [18].

Analysis of the total number of spermatogonia showed that the number of animals of the experimental group decreased, compared with control, on 6.31%.

Decrease in this indicator, obviously, is connected with the beginning of a new wave of spermatogenesis to the period of puberty, which is more intense and terminated not by apoptosis, and the formation of healthy spermatozoa [19].

As a result of the studies noted a decrease in the number of spermatozoa in the lumen of the convoluted seminiferous tubule in 26.70% (p≤0.05) compared to control, reducing the amount of data cells is, apparently, a reflection of changes in the number of spermatogonia, spermatocyte, and spermatids.

The most important quantitative indicator of the generative activity of the testis is the index of spermatogenesis, showing the number of generations of spermatogenic cells in the wall of the convoluted seminiferous tubules [20]. The index of spermatogenesis is one of the most important indicators of the state of spermatogenic layer [21,22]. Marked decline in the index of spermatogenesis in the experimental group compared with control at 10.24%. Decrease in this indicator always indicates disturbances of spermatogenesis and decreased functional activity of seminal gland [22-24].

The increase in the index of maturation of 20.00% (p≤0.05), index maati-cal activity on 23.08% (p≤0.05), and germinative index by 31.79% (p≤0.05) shows the prevalence of young cells on improvements in mature and delay the maturation of male germ cells [24].

Another indicator that has a significant impact on fertility is sperm viability. The increase in the number of dead sperm in the homogenate of the appendages of the male sex glands in sexually mature animals of the experimental groups compared to intact control animals on 60.68% (p≤0.05). Hence, in intact animals of the control group the content of dead cells in 1 ml of suspension amounted 11.56% (p≤0.05) from the total number of cells, and in animals of the experimental group – 59.54% (p≤0.05). Analysis of the number of pathological forms of spermatozoa was acknowledged significant increase in the number of degenerative forms after exposure to lead acetate. During the survey, it was found that in normal spermatozoa in the lumen of the convoluted seminiferous tubule are groups of 6-8 around the contour of the lumen. In mature spermatozoa smears have a clear separation into its component parts: Head, neck, and tail. Most of the sperm head has the shape of a hook. After 7 days of exposure of lead, acetate marked disorderly arrangement of spermatozoa in the lumen of the tubule. Reduced size and shape of the sperm head. On history, path observed the cliffs tailings and agglutination of sperm. Discovered convoluted seminiferous tubules in the lumen which there was no spermatozoa.

Thus, in animals of the experimental group had significantly reduced both the quantitative and qualitative characteristics of spermatozoa.

## Conclusions

The results of the studies testify to the negative impact of lead acetate in the course of the process of spermatogenesis in male albino rats:

1. Found that when exposed to lead acetate reduced production of all populations of spermatogenic cells and especially their mature forms – spermatids and spermatozoa
2. Reduces the number of stem cells – spermatogonia, which is an adverse prognostic factor of the process of spermatogenesis
3. After exposure to lead acetate in preparations, there are lack spermatozoa heads in the majority and change their shape
4. Reduced index of spermatogenesis and the index of relaxation (tension of spermatogenesis), in comparison with control, which indicates a reduction of the functional activity of the testes. Simultaneously with this increase, in comparison with the control index ripening, index meiotic activity, and germinative index that indicates the prevalence of young cells of a mature and delayed ripening of male germ cells.
5. Lead acetate reduces the productivity of the seminal glands, resulting in a decrease in the concentration of spermatozoa in suspension, and their viability.

## Authors’ Contributions

OSS and NAD: Designed the research work and provided the technical guidance. NAD: Conducted the research work. OSS: Provided necessary help for animal experimentation. NAD: Carried out all the statistical analysis. Both authors participated in the study design, research work, discussion, draft, and revision of the manuscript. Both authors read and approved the final manuscript.
